# Segregation of *LIPG*, *CETP*, and *GALNT2* Mutations in Caucasian Families with Extremely High HDL Cholesterol

**DOI:** 10.1371/journal.pone.0037437

**Published:** 2012-08-27

**Authors:** Ian Tietjen, G. Kees Hovingh, Roshni R. Singaraja, Chris Radomski, Amina Barhdadi, Jason McEwen, Elden Chan, Maryanne Mattice, Annick Legendre, Patrick L. Franchini, Marie-Pierre Dubé, John J. P. Kastelein, Michael R. Hayden

**Affiliations:** 1 Xenon Pharmaceuticals Inc., Burnaby, Canada; 2 Department of Vascular Medicine, Academic Medical Center, University of Amsterdam, The Netherlands; 3 Centre for Molecular Medicine and Therapeutics, University of British Columbia, Vancouver, Canada; 4 Montreal Heart Institute, Montréal, Canada; Universitätsklinikum Schleswig-Holstein - Campus Luebeck, Germany

## Abstract

To date, few mutations are described to underlie highly-elevated HDLc levels in families. Here we sequenced the coding regions and adjacent sequence of the *LIPG*, *CETP*, and *GALNT2* genes in 171 unrelated Dutch Caucasian probands with HDLc≥90th percentile and analyzed segregation of mutations with lipid phenotypes in family members. In these probands, mutations were most frequent in *LIPG* (12.9%) followed by *GALNT2* (2.3%) and *CETP* (0.6%). A total of 6 of 10 mutations in these three genes were novel (60.0%), and mutations segregated with elevated HDLc in families. Interestingly, the *LIPG* mutations N396S and R476W, which usually result in elevated HDLc, were unexpectedly found in 6 probands with low HDLc (i.e., ≤10th percentile). However, 5 of these probands also carried mutations in *ABCA1*, *LCAT*, or *LPL*. Finally, no *CETP* and *GALNT2* mutations were found in 136 unrelated probands with low HDLc. Taken together, we show that rare coding and splicing mutations in *LIPG*, *CETP*, and *GALNT2* are enriched in persons with hyperalphalipoproteinemia and segregate with elevated HDLc in families. Moreover, *LIPG* mutations do not overcome low HDLc in individuals with *ABCA1* and possibly *LCAT* and *LPL* mutations, indicating that *LIPG* affects HDLc levels downstream of these proteins.

## Introduction

Coronary artery disease (CAD) is the leading cause of mortality in the industrialized world [1], demonstrating an urgent and unmet need to develop new therapies that reduce risk for CAD [2]. Epidemiological studies show that increased plasma HDLc confers significant protection against CAD [3], [4], in part by elevating reverse cholesterol transport and its anti-inflammatory properties [5], [6]. In contrast to common SNP alleles that associate with very small lipid changes in large populations [7], gene mutations that are extremely rare in the general population and result in major elevations in HDLc levels are more likely to mimic the effects of drugs such as CETP inhibitors that have major inhibitory effects on protein function [8], [9].

The successful identification of these rare mutations is often precluded by their low frequency in populations, which generally limits the power to test for associations [10]. Instead, they are often validated by assessing their segregation in families with apparent Mendelian forms of extreme HDLc traits [11]–[17]. However, while this approach has been very successful in identifying mutations in families with extremely low HDLc, few studies to date report mutations that lead to highly-elevated HDLc in families [10], [18].

HDLc turnover is significantly controlled by the gene products of *LIPG* and *CETP* [19]. *LIPG* encodes endothelial lipase (EL), a major regulator of HDL phospholipid catabolism. Loss-of-function of EL significantly increases HDLc levels in mice and humans, potentially through reduced catabolism of large, lipid-rich HDL particles [17], [20]–[22], while loss-of-function mutations in families do not affect other lipid measures [17], [23]. *CETP* encodes cholesteryl ester transfer protein, which exchanges triglycerides from VLDL and LDL particles for cholesterol esters from HDL and selectively enhances liver HDL cholesterol ester uptake [24]. In families, loss-of-function *CETP* mutations that inhibit cholesterol ester transfer result in larger HDL particles, reduced catabolism, and increased serum HDLc, in addition to reduced LDLc [11], [16], [25], [26].

Another strong candidate for HDLc regulation in humans is *GALNT2*, which regulates O-linked oligosaccharide biosynthesis [27]. Common *GALNT2* SNPs associate with very small (e.g., ∼1–2%) changes in HDLc and triglycerides in large genome-wide association studies [7]. Moreover, *Galnt2* over-expression in mouse liver reduces HDLc, while shRNA-based *Galnt2* knockdown increases HDLc [7]. More recently, a rare familial *GALNT2* mutation was observed to underlie accelerated postprandial triglyceride clearance and increased unsialylated apolipoprotein C-III (APOC3) levels, which in turn associated with elevated lipoprotein lipase (LPL) activity [28]. Mutations in both APOC3 and LPL also lead to large changes in HDLc [13], [29], [30], further supporting that mutations in *GALNT2* may also affect HDLc in humans.

Here we performed a family-based study to assess the segregation of rare mutations with elevated HDLc levels. We first identified mutations by sequencing the coding regions and adjacent UTR and intronic sequences of *LIPG*, *CETP*, and *GALNT2* in 171 unrelated individuals with extremely high HDLc (defined here as HDLc≥90^th^ percentile adjusted for age and gender) [31], and compared this to sequence data from 136 persons with extremely low HDLc (HDLc≤10^th^ percentile). We then assessed the family members of probands with rare mutations to determine the associations of these mutations with lipid phenotypes.

## Materials and Methods

### Patients

We identified 171 unrelated probands of Dutch Caucasian ancestry with HDLc≥90^th^ percentile and 136 unrelated Dutch Caucasian probands with HDLc≤10^th^ percentile (based on age- and sex-specific Lipid Research Clinic data and as described previously) [31], [32] with no other abnormal lipid measures. We also studied 199 family members of 29 total probands with mutations. The study protocol was approved by the Ethics Committees of the Academic Medical Center, Amsterdam. All subjects provided written informed consent. Lipoprotein measurements were performed on fresh plasma as described [33]. Cholesterol and triglyceride levels were determined in total plasma and plasma at density d<1.006 g/mL obtained after preparative ultracentrifugation, before and after precipitation with dextran manganese.

### DNA sequencing and data analysis

The *LIPG*, *CETP*, and *GALNT2* coding regions and adjacent intron and UTR sequence as defined in human genome hg18 were sequenced from genomic DNA in all probands using either standard fluorescent dye terminator chemistry (Seqwright, Houston TX) or next generation paired-end read sequencing (Illumina, San Diego CA). For standard sequencing, DNA primers were designed to flank *LIPG*, *CETP*, and *GALNT2* exons and adjacent intron and UTR sequence and sequenced bidirectionally. Primer sequences are listed in Table S7. Sequence changes were identified using Sequencher v4.7 (Ann Arbor, MI) and confirmed in dbSNP build 130 and 1000Genomes November 2010 data release or by sample resequencing. For next generation sequencing, sequence changes were identified by alignment of sequence data to the human genome (NCBI Build 36.1) using CASAVA v.1.7 software (Illumina, San Diego CA). Sequence changes predicted to be damaging to protein function as defined below were then confirmed by standard sequencing as described above. In all cases, data analysis was performed by individuals who were blinded to the phenotypes of sequenced individuals. Reference DNA/Protein sequences used for data analysis include NM_006033.2/NP_006024 for LIPG, NM_000078.2/NP_000069 for CETP, NM_004481.3/NP_004472 for GALNT2, NM_005502.2/NP_005493 for ABCA1, NM_000039.1/NP_000030 for APOA1, NM_000229.1/NP_000220 for LCAT, and NM_000237.2/NP_000228 for LPL.

Mutations were defined as any sequence change that is predicted to be damaging to protein function as determined by Polyphen-2 (http://genetics.bwh.harvard.edu/pph2/) and/or Spliceview (http://zeus2.itb.cnr.it/~webgene/wwwspliceview_ex.html) *in silico* algorithms [34], [35]. Vertebrate sequence conservation of mutations was determined using the conservation tracks in the UCSC Genome Bioinformatics Human Genome Browser Gateway (http://genome.ucsc.edu/).

### Segregation analysis of mutations in families

Family members of probands with mutations were genotyped using primers and standard sequencing techniques described above. Lipid parameters of mutation carriers were compared to first-degree relative controls, defined as all parents, siblings, and children of persons with a mutation that do not carry a known mutation themselves.

### Statistical analyses

In total probands, single mutation burden effects for each gene were assessed by Fisher's exact test. Where appropriate, combined mutation burden effects for each gene were assessed by C-alpha test [36] and cohort allelic sum test (CAST) [37]. Combined mutation burden effects for all genes were also assessed by combined multivariate and collapsing (CMC) test [38]. In contrast to the collapsing and CMC methods, the C-alpha test allows for the joint presence of risk and protective variants. The CMC method is used for multiple predefined groups and expands on the collapsing method for a single group of markers. Groups were defined as genes for the present purpose. Fisher exact test was performed when only a single variant-test is considered. For all tests, a p value of 0.05 or less was considered significant.

In total families, significance of mutations with lipid profiles in carrier individuals vs. first-degree relative controls was determined by stratified conditional logistic regression. The matched sets (strata) were defined by the family number. For *CETP* and *GALNT2*, an exact stratified conditional logistic regression was performed due to the small sample size. Results of each lipid profile were adjusted for age and sex. A p value of 0.05 or less was considered significant.

## Results

### LIPG, CETP, and GALNT2 mutations in probands with high HDLc

**Figure 1:**
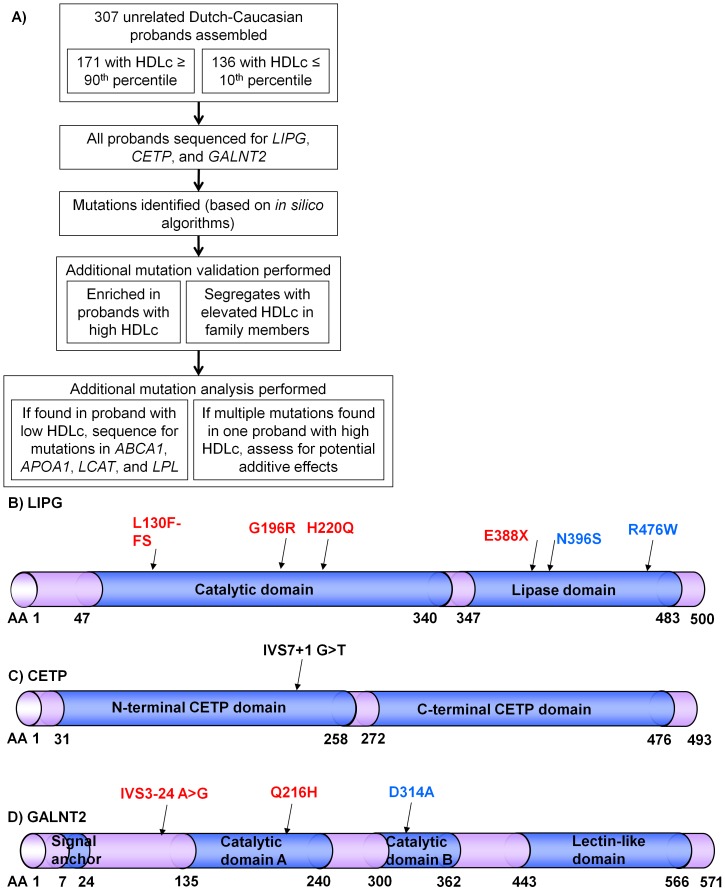
Mutations identified in probands with high or low HDLc. A) Overview of the study design. **B–D**) Predicted mutation effects on **B**) LIPG, **C**) CETP, and **D**) GALNT2 proteins. Red sequence changes are novel, blue are previously described [17], [23], [28], and black is previously described in a subset of this cohort [16].

An overview of the study design is shown in Figure 1A. We assembled 171 unrelated Dutch probands with HDL≥90^th^ percentile (Table 1) and sequenced the coding regions and adjacent UTR and intronic sequence of *LIPG*, *CETP*, and *GALNT2*. Mutations were defined as any sequence change that is predicted to be damaging to protein function as determined by Polyphen-2 and/or Spliceview *in silico* algorithms [34], [35]. In all individuals, mutations were most frequently identified in *LIPG* (22 of 171, 12.9%) but were rarely found in *CETP* (1 of 171, 0.6%) or *GALNT2* (4 of 171, 2.3%) (Table 2).

**Table 1:** Demographics of unrelated probands with extreme HDLc.

Measure	HDLc percentile	
	≥90^th^	≤10^th^
Total assessed	171	136
Age (y)^a^	53.6 (14.1)	51.8 (14.2)
Male individuals^b^	85 (49.7%)	88 (64.7%)
Total cholesterol (mmol/L)^a^	5.94 (1.06)	4.48 (1.36)
Triglycerides (mmol/L)^a^	0.88 (0.44)	1.68 (1.01)
HDLc (mmol/L)^c^	2.23 (2.16–2.31)	0.71 (0.68–0.71)
LDLc (mmol/L)^a^	3.30 (0.92)	3.01 (1.23)
BMI (kg/m2)^a^	24.0 (3.1)	27.0 (4.6)

a , Average (SD);

b , N (%);

c , Average (95% confidence interval).

**Table 2:** Mutation frequencies and burden effects in probands.

Gene	Mutations	N (%)		p value			
		≥90th	≤10th	C-alpha	CAST	CMC	Fisher's
N sequenced		171	136				
LIPG	Total	22 (19.9%)	3 (2.2%)	2.7*10^−3^	5.5*10^−4^		
	N396S	11 (6.4%)	2 (1.5%)				**0.04**
	R476W	6 (3.5%)	1 (0.7%)				0.14
CETP	Total	1 (0.6%)	0 (0.0%)				0.55
GALNT2	Total	4 (2.3%)	0 (0.0%)	0.35	0.13		
All genes	Total	25 (14.6%)^a^	3 (2.2%)	2.6*10^−3^	9.9*10^−5^	3.0*10^−3^	
	Novel	8 (4.7%)	0 (0.0%)	0.30	9.9*10^−3^	**0.04**	

a , Two probands have rare *LIPG*+*GALNT2* variants.

If mutations in *LIPG*, *CETP*, and *GALNT2* underlie elevated HDLc, then they should be less frequent or absent in control probands with the opposite phenotype (i.e., extremely low HDLc). We therefore sequenced the same coding regions and adjacent sequence of these genes in 136 unrelated probands with HDLc≤10^th^ percentile (Table 1). No *CETP* or *GALNT2* mutations were found in probands with low HDLc (Table 2). In contrast, three probands with low HDLc were unexpectedly observed to carry the previously-described HDLc-raising *LIPG* mutations N396S or R476W [17], [23], raising the possibility of additional genetic factors that may cause low HDLc in the presence of these mutations (see below). Total *LIPG* mutations were significantly enriched in probands with elevated HDLc (e.g., p = 2.7*10^−3^ by C-alpha test; Table 2). In contrast, the frequencies of these very rare *CETP* and *GALNT2* mutations did not reach statistical significance in our small and likely underpowered cohort of total probands, indicating the need to test the segregation of these mutations with elevated HDLc in family members.

The predicted functional consequences of all mutations are shown in Figure 1B–D and summarized in Table 3. Of 10 total mutations identified in three genes, 4 of 6 *LIPG* mutations and 2 of 3 *GALNT2* mutations are not reported in previous literature, dbSNP, or 1000Genomes (Table 3). Notably, the novel *LIPG* mutation E388X was identified in two Dutch probands who have no additional sequence variation across all *LIPG* exons and adjacent sequence, implying a shared ancestral haplotype of at least 24.5 kb. Moreover, the *GALNT2* Q216H mutation was also identified in two Dutch probands, and segregation analysis of common SNPs in close proximity to *GALNT2* Q216H in family members indicated a shared ancestral haplotype of at least 7.2 kb (data not shown). No novel mutations were detected in 136 individuals with HDLc≤10^th^ percentile (e.g., p = 9.9*10^−3^ by CAST and p = 0.04 by CMC test; Table 2).

**Table 3:** *LIPG*, *CETP*, and *GALNT2* mutations.

Gene	Mutation		Novel?	# of occurrences		Vertebrate conservation	Predicted effect
	Position (hg18)	Effect		HDLc≥90th %ile	HDLc≤10th %ile		
LIPG	Chr18:45347918 C>del	L130F-FS	Yes	1	0	Completely conserved	Truncates protein
	Chr18:45355751 G>A	G196R	Yes	1	0	Completely conserved	Probably damaging (Polyphen)
	Chr18:45355825 C>G	H220Q	Yes	1	0	Completely conserved	Probably damaging (Polyphen)
	Chr18:45362846 G>T	E388X	Yes	2	0	Completely conserved	Truncates protein
	Chr18:45363953 A>G	N396S	No [23]	11	2	Completely conserved	Possibly damaging (Polyphen)
	Chr18:45367163 C>T	R476W	No [17]	6	1	Lys, Gln tolerated	Possibly damaging (Polyphen)
CETP	Chr16:55562795 G>T	IVS7+1 G>T	No [16]	1	0	Completely conserved	Abolishes splice donor site (Spliceview)
GALNT2	Chr1:228438359 A>G	IVS3-24 A>G	Yes	1	0	Completely conserved	Introduces ectopic splice acceptor site (Spliceview)
	Chr1:228445715 A>T	Q216H	Yes	2	0	Completely conserved	Possibly damaging (Polyphen)
	Chr1:228452861 A>C	D314A	No [28]	1	0	Completely conserved	Possibly damaging (Polyphen)

If rare, deleterious mutations in these genes are indeed identified to be enriched in probands with elevated HDLc, then non-deleterious DNA changes should occur with similar frequencies in both probands with extremely high or low HDLc. We therefore assessed the minor allele frequencies for all additional SNPs identified in *GALNT2*, in addition to all additional coding SNPs in *LIPG* and *CETP*, in a representative subset of 55 probands with HDLc≥90^th^ and 55 probands with HDLc≤10^th^ percentiles for which standard fluorescent dye terminator-based sequencing data were available (Tables S1 and S2). Of 15 additional SNPs found in *GALNT2*, none were predicted to be nonsynonymous or affect splicing with the exception of a known V554M polymorphism that is predicted to be functionally benign. Furthermore, no minor alleles for any *GALNT2* SNP were observed to be significantly enriched in either proband population (Table S1). In *LIPG*, the synonymous SNP S21S and the previously-described nonfunctional T111I polymorphism [17] were identified, neither of which were significantly associated with high or low HDLc (Table S2). In *CETP*, two benign coding SNPs (F287F and I422V) were also identified with no significant minor allele associations with elevated HDLc (Table S2). Finally, for *LIPG* and *CETP*, no additional noncoding SNPs were identified with predicted deleterious effects on splicing (data not shown). Taken together, these observations further indicate that only *LIPG*, *CETP*, and *GALNT2* mutations with predicted deleterious effects, but not non-deleterious sequence changes, are distinctly enriched in individuals with elevated HDLc.

Taken together, 25 of 171 (14.6%) total probands with HDLc≥90^th^ percentile have mutations in ≥1 gene (Table 2), with 6 of 10 total mutations being novel (60.0%; Table 3). When total mutations across all sequenced exons and adjacent intronic sequence are considered, we found 1 *LIPG* mutation per 673 base pairs (bp) of sequence (6 mutations found in 4,036 total sequenced bp), 1 *CETP* mutation per 5,292 bp (1 mutation in 5,292 total sequenced bp), and 1 *GALNT2* mutation per 1,488 bp (3 mutations in 4,465 total sequenced bp).

### Mutation segregation with elevated HDLc in families

**Figure 2:**
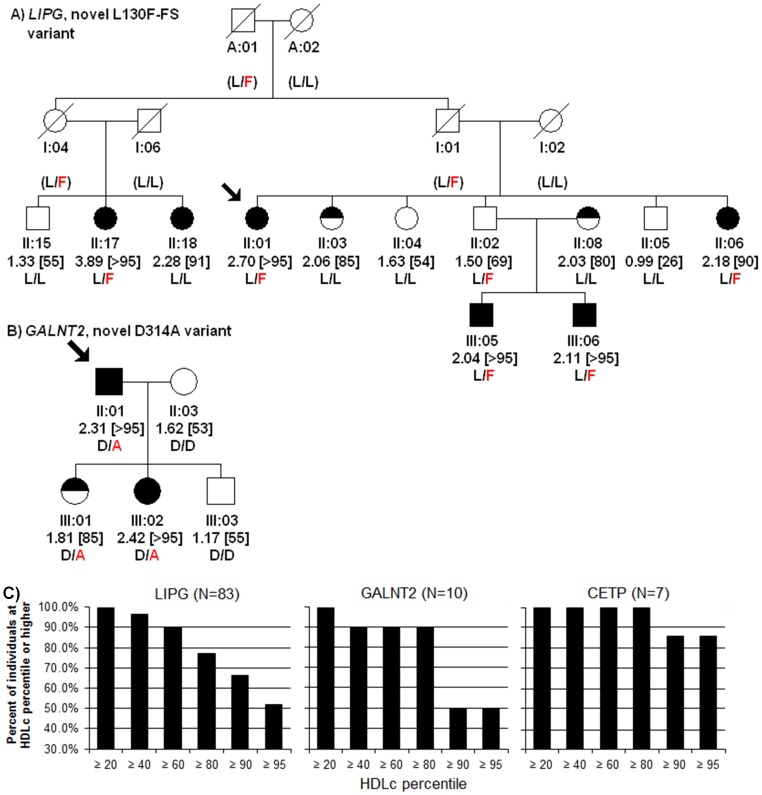
Representative segregation of mutations in families. **A, B**) Segregation of (**A**) *LIPG* L130F-FS and (**B**) *GALNT2* D314A with elevated HDLc. For each individual, the individual ID, HDLc (in mmol/L) plus [HDLc percentile], and genotype are shown. Squares, Males; Circles, Females; Arrow, proband. Filled shape, HDLc≥90^th^ percentile; half-filled, HDLc between 80–89^th^ percentiles; empty shape, HDLc<80^th^ percentile. Slash = deceased. **C**) Percent of individuals in families with mutations at given HDLc percentiles or higher.

For 22 total probands with HDLc≥90^th^ percentile and single mutations in *LIPG*, *CETP*, or *GALNT2*, we genotyped all available family members and assessed lipid phenotypes (Figure 2A–B; Figure S1; Table 4). For *LIPG* and *CETP*, significant segregation of mutations was observed with elevated HDLc, further supporting that these mutations are likely to be deleterious and confer large changes on HDLc. For example, assessment of 83 total *LIPG* mutation carriers in families revealed an average 0.33 mmol/L elevated HDLc compared to 80 first-degree relative controls that do not carry any known mutations (+19.3%, p = 7.47*10^−6^). Moreover, 7 *CETP* mutation carriers had an average 0.96 mmol/L increased HDLc vs. 9 first-degree relative controls (+62.3%, p = 0.027). For *GALNT2*, an average but borderline significant 0.52 mmol/L HDLc increase was observed in 10 total *GALNT2* mutation carriers vs. 11 first-degree controls (+34.2%, p = 0.093), suggesting that either *GALNT2* mutations have weaker or more variable effects than *LIPG* and *CETP* mutations on HDLc elevation and/or these families have insufficient power for detecting significant *GALNT2* mutation associations with increased HDLc. In contrast, no significant changes in triglycerides, LDLc, or BMI were observed for mutations of any gene.

**Table 4:** Phenotypes of family members with single *LIPG, CETP, and GALNT2* mutations.

Gene	Measure	1st-degree controls	Mutation carriers	% change	p value
LIPG	Total assessed	80	83		
	Age (y)^a^	44.5 (19.0)	43.9 (20.7)		0.371
	Male individuals^b^	32 (40.0%)	46 (55.4%)		0.074
	Total cholesterol (mmol/L)^a^	5.40 (1.32)	5.83 (1.41)	7.9%	0.224
	Triglycerides (mmol/L)^a^	1.05 (0.72)	1.02 (0.64)	−2.9%	0.338
	HDLc (mmol/L)^a^	1.71 (0.48)	2.04 (0.64)	19.3%	7.47*10^−6^
	LDLc (mmol/L)^a^	3.21 (1.22)	3.32 (1.21)	3.4%	0.368
	BMI (kg/m2)^a^	23.6 (4.5)	22.7 (3.2)	−3.4%	0.191
CETP	Total assessed	9	7		
	Age (y)^a^	42.8 (15.4)	34.3 (19.4)		0.349
	Male individuals^b^	4 (44.4%)	2 (28.6%)		0.632
	Total cholesterol (mmol/L)^a^	6.05 (0.72)	5.61 (0.70)	−7.3%	0.378
	Triglycerides (mmol/L)^a^	2.11 (1.35)	1.11 (0.29)	−47.4%	0.135
	HDLc (mmol/L)^a^	1.54 (0.31)	2.50 (0.45)	62.3%	**0.027**
	LDLc (mmol/L)^a^	3.53 (0.65)	2.60 (0.33)	−26.5%	0.135
	BMI (kg/m2)^a^	24.5 (3.0)	21.6 (2.6)	−12.0%	0.250
GALNT2	Total assessed	11	10		
	Age (y)^a^	31.5 (18.3)	43.2 (15.1)		0.170
	Male individuals^b^	8 (72.7%)	4 (40.0%)		0.220
	Total cholesterol (mmol/L)^a^	5.25 (0.95)	6.15 (1.20)	17.1%	0.500
	Triglycerides (mmol/L)^a^	1.38 (1.30)	1.21 (0.84)	−12.3%	0.879
	HDLc (mmol/L)^a^	1.52 (0.29)	2.04 (0.64)	34.2%	0.093
	LDLc (mmol/L)^a^	3.09 (0.75)	3.55 (1.02)	14.9%	0.937
	BMI (kg/m2)^a^	21.4 (3.1)	23.6 (2.1)	10.3%	0.400

a , Average (SD) shown;

b , N (%) shown.

We next estimated the relative expressivity of these mutations to elevate HDLc (Figure 2C). In total, only 55 of 83 individuals with only *LIPG* mutations manifested HDLc≥90^th^ percentile (66.3%), while 5 of 10 (50.0%) *GALNT2* mutation carriers exhibited HDLc≥90^th^ percentiles. In contrast, 6 of 7 *CETP* mutation carriers had HDLc≥90^th^ percentile (85.7%), suggesting that these *CETP* mutations may confer stronger average effects on HDLc elevation than *LIPG* mutations, which in turn may confer stronger effects than *GALNT2* mutations.

### Low HDLc in individuals with mutations in LIPG plus ABCA1, LCAT, or LPL

As described above, although *LIPG* mutations were significantly more prevalent in probands with high HDLc (e.g., p = 2.7*10^−3^ by C-alpha test, Table 2), 3 probands with low HDLc were unexpectedly observed to carry the previously-described HDLc-raising *LIPG* mutations N396S or R476W [17], [23]. Genotyping of an independent cohort of 34 unrelated, Dutch-Caucasian probands with HDLc≤10^th^ percentile (data not shown) identified 3 more carriers of N396S or R476W, for a total of 6 individuals with these LIPG mutations and low HDLc (4 with N396S and 2 with R476W).

As the *LIPG* mutations N396S and R476W generally associate with elevated HDLc both here and elsewhere [17], [23], we asked whether additional genetic factors may cause low HDLc in the presence of these *LIPG* mutations. We therefore sequenced these 6 probands for *ABCA1*, *APOA1*, *LCAT*, and *LPL*, which are all well-established to harbor mutations that cause low HDLc [13], [14], [39], [40]. Strikingly, 5 of 6 probands with *LIPG* mutations and low HDLc also carried mutations in *ABCA1* (3 of 6), *LCAT* (1 of 6), and *LPL* (1 of 6, Table 5). All mutations found in these genes are predicted to be damaging by Polyphen-2 or Spliceview [34], [35] with the exception of the *LPL* mutation N296S, which is previously reported to underlie low HDLc [13].

**Table 5:** Additional mutations in individuals with *LIPG* mutations and low HDLc.

Gene	Mutation		Novel?	Vertebrate conservation	Predicted effect
	Position (hg18)	Effect			
ABCA1	Chr9:106631215 T>A	M640L	No [47]	Completely conserved	Possibly damaging (Polyphen-2)
	Chr9:106619433 C>G	IVS24+1G>C	No [15]	Completely conserved	Abolishes splice donor site (Spliceview)
	Chr9:106618441 G>A	S1181F	No [48]	Completely conserved	Probably damaging (Polyphen-2)
LCAT	Chr16:66534158 G>A	T147I	No [12]	Completely conserved	Possibly damaging (Polyphen-2)
LPL	Chr8:19857809 A>G	N318S	No [13]	Ser tolerated	Reduced HDLc (Reymer et al., 1995)

**Figure 3:**
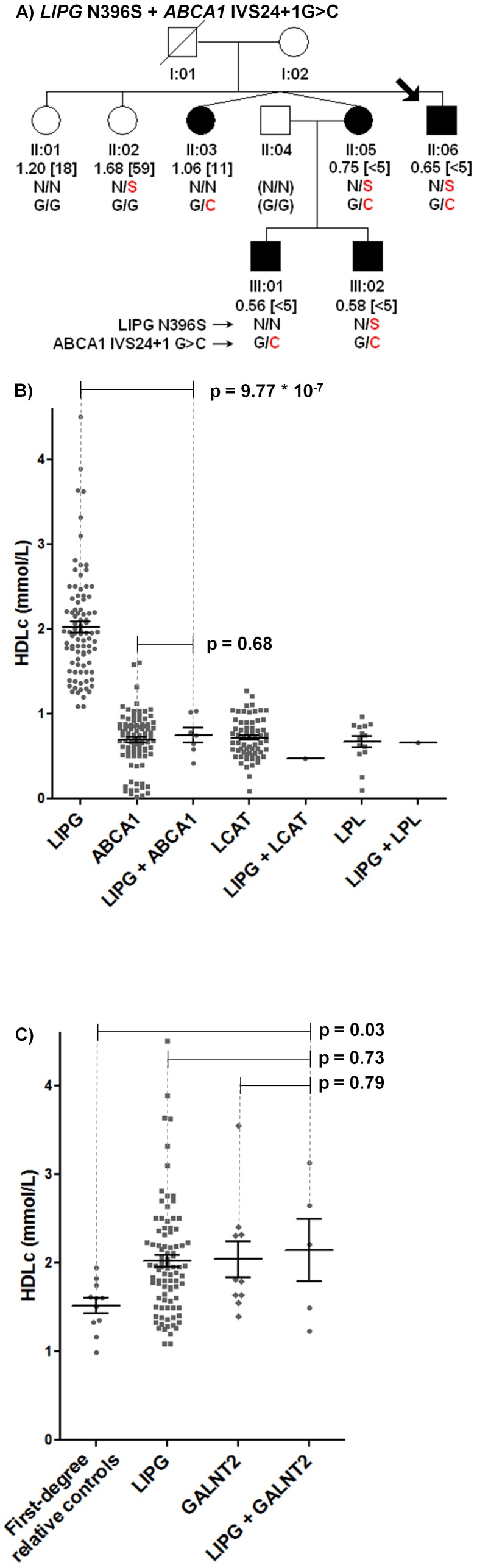
Characterization of probands and families with multiple mutations. **A**) Representative segregation of *LIPG* N396S and *ABCA1* IVS24+1G>C mutations. Filled shape, HDLc<15^th^ percentile; empty shape, HDLc≥15^th^ percentile. **B**) HDLc levels of individuals with *LIPG*+/−*ABCA1*, *LCAT*, or *LPL* mutations. Individuals with *LIPG* mutations alone are described in Table 4. Individuals with single *ABCA1*, *LCAT*, and *LPL* mutations are from an independent Dutch-Caucasian population (Tables S1, S2, and S3 and data not shown). **C**) HDLc levels of individuals with mutations in *LIPG*, *GALNT2*, or *LIPG+GALNT2*. Individuals with single *LIPG* or *GALNT2* mutations are described in Table 4.

In addition, family members with the *LIPG* N396S mutation consistently manifested low HDLc in the presence of a known *ABCA1* mutation (Figure 3A; Figure S2) [15]. In total, 7 individuals with *LIPG+ABCA1* mutations from 3 families had an average 1.29 mmol/L lower HDLc vs. 83 unrelated individuals with only *LIPG* mutations (0.75 vs. 2.04 mmol/L, −63.2%, p = 9.77*10^−7^), but virtually no difference in HDLc vs. 93 independent, unrelated Dutch Caucasian individuals with *ABCA1* mutations (0.75 vs. 0.70 mmol/L, +7.1%, p = 0.68; Figure 3B; Table S3). Moreover, one individual with both *LIPG*+*LCAT* mutations had 1.57 mmol/L lower HDLc vs. individuals with only *LIPG* mutations (0.47 mmol/L; −76.9%; Table S4). Similarly, one individual with both *LIPG+LPL* mutations had 1.38 mmol/L lower HDLc vs. individuals with only *LIPG* mutations (0.66 mmol/L; −67.6%; Table S5). Thus low HDLc in 5 of 6 probands with established HDLc-raising mutations in *LIPG* can be attributed to mutations in *ABCA1*, *LCAT*, and *LPL*.

### No further HDLc elevation in individuals with LIPG+GALNT2 mutations

We also identified 2 probands and 3 family members with both *GALNT2* Q216H and either *LIPG* N396S or R476W. These individuals had an average 0.62 mmol/L increased HDLc vs. 11 first-degree relative controls with no mutations (+40.8%, p = 0.032; Figure 3C; Table S6). However, no significant differences in HDLc or other lipid measures were observed in individuals with both *LIPG+GALNT2* mutations when compared to individuals with mutations in either *LIPG* alone (0.11 mmol/L increased HDLc vs. 89 individuals with *LIPG* mutations, +5.9%, p = 0.67) or *GALNT2* alone (0.10 mmol/L increased HDLc vs. 10 individuals with *GALNT2* mutations, +4.9%, p = 0.79), raising the possibility that mutations in *GALNT2* and *LIPG* may be redundant with respect to HDLc elevation.

## Discussion

To date, few mutations have been described to lead to large changes in HDLc elevation in families [10], [18]. Here we performed a family-based study by initially sequencing the coding regions and adjacent sequence of *LIPG*, *CETP*, and *GALNT2* in a series of probands with extreme HDLc levels and searching for rare mutations with expected large effects on HDLc elevation. Mutations were then further validated by genotyping available family members of each proband and by assessing segregation with extreme HDLc phenotypes. In 171 probands with HDLc≥90^th^ percentile, mutations were most frequently identified in *LIPG* (22 of 171, 12.9%), followed by *GALNT2* (4 of 171, 2.3%) and *CETP* (1 of 171, 0.6%), with significantly increased total and *LIPG* mutations in probands with HDLc≥90^th^ percentile when compared to 136 probands with HDLc≤10^th^ percentile. We also found no *CETP* or *GALNT2* mutations in probands with HDLc≤10^th^ percentile. Interestingly, and in contrast to these results, a recent sequencing study of 64 Thai individuals with hyperalphalipoprotenemia [41] identified 6 *CETP* mutations, *2* mutations in hepatic lipase, and no *LIPG* mutations, suggesting that enrichment of HDLc-elevating mutations in distinct genes may be dependent on geographic origin, a possibility which warrants further investigation in additional cohorts of different ancestries.

When assessed in family members, *LIPG*, *CETP*, and *GALNT2* mutations described here segregated respectively with average HDLc elevations of 0.33, 0.66, and 0.52 mmol/L in carriers compared to first-degree relative controls, with no other significant changes in lipids. Segregation of *LIPG* and *CETP* mutations with elevated HDLc in families was significant, while *GALNT2* mutations were borderline significant. Notably, mutations in *LIPG* (E388X) and *GALNT2* (Q216H) were each found in 2 of 171 Dutch probands with HDLc≥90^th^ percentile (1.2%). Evidence of founder effects for these mutations was obtained from shared ancestral haplotypes in probands and family members, suggesting that these two mutations may each potentially be present in ∼1% of HDLc≥90^th^ percentile in the Dutch population. Finally, we show that the large majority (∼85%) of probands with HDLc≥90^th^ percentile in this cohort have no known causes of elevated HDLc, thereby emphasizing the opportunity to discover novel genetic factors of hyperalphalipoproteinemia.

Recently, the *GALNT2* D314A loss-of-function mutation was observed in humans to underlie non-sialyation of APOC3, which in turn led to increased LPL activity [28]. In that study, only 2 of 243 subjects with HDLc≥95^th^ percentile had *GALNT2* mutations, emphasizing that *GALNT2* mutations are exceedingly rare. Similarly, we identify *GALNT2* mutations in only 4 of 171 probands with HDLc≥90^th^ percentile while identifying only 2 additional novel mutations. Although *GALNT2* mutations in families associated with a large (average 0.52 mmol/L) elevation in HDLc in this study, this trend was borderline statistically significant, suggesting that either *GALNT2* mutations have weaker or variable effects on HDLc elevation than *LIPG* or *CETP* mutations and/or these families have insufficient power for detecting a significant association. Regardless, our observations are consistent with mis-expression studies in mice, where *Galnt2* mRNA levels are inversely proportional to HDLc levels [7]. Moreover, loss-of-function mutations in both *APOC3* and *LPL* result in large increases and reductions in HDLc, respectively [13], [29], [30], which further support a role for *GALNT2* loss-of-function mutations in HDLc elevation. We also observed that individuals with both *GALNT2* and *LIPG* mutations had HDLc levels similar to individuals with mutations in either *GALNT2* or *LIPG* alone, raising the possibility that mutations in *GALNT2* and *LIPG* may be redundant with respect to HDLc elevation. Notably, we also did not observe a significant reduction in triglycerides in family members with *GALNT2* mutations in this study. However, triglyceride levels in this study were measured in the fasting state, whereas triglyceride effects in *GALNT2* mutation carriers becomes more apparent following oral fat challenge [28]. Additional phenotypic analysis of these and additional individuals with rare *GALNT2* mutations is warranted to elucidate the exact mechanisms by which these mutations may lead to elevated HDLc in humans.

We unexpectedly observed the HDLc-elevating *LIPG* N396S and R476W mutations in 6 probands with HDLc≤10^th^ percentile. However, 5 of these probands also carried mutations in *ABCA1*, *LCAT*, or *LPL* [13], [14], [39]. Moreover, family members with *LIPG* plus *ABCA1* mutations consistently manifested phenotypes that resemble those of individuals with mutations in *ABCA1* alone. These observations indicate that *LIPG* N396S and R476W do not overcome the loss-of-function effects of *ABCA1*, *LCAT*, and *LPL* mutations in these cohorts. They also suggest that patients with mutations in these genes are less likely to respond to HDLc-raising therapeutic strategies that target antagonism of *LIPG*.

How might *ABCA1*, *LCAT*, and *LPL* mutations overcome the HDLc-elevating effects of *LIPG* mutations? *ABCA1* encodes ATP-binding cassette transporter A1, which is a cell membrane transporter that regulates cholesterol efflux from tissues [19]. LCAT, in turn, converts free cholesterol to cholesterol esters as part of mature, spherical HDL particle formation [39]. Finally, *LPL* encodes a triglyceride hydrolase that regulates delivery of apolipoproteins and phospholipids to HDL [42], [43]. ABCA1-mediated cholesterol efflux leads to lipidation and formation of pre-beta HDL particles, which mature into large spherical HDL particles via LCAT and LPL-mediated mechanisms. In contrast, EL is involved in downstream HDL phospholipid catabolism and thus acts on particles that are already modified by ABCA1, LCAT, and LPL. The inability of some *LIPG* mutations to overcome the effects of *ABCA1*, *LCAT*, and *LPL* mutations is therefore consistent with the sequential roles of the respective gene products in HDL metabolism. These studies exquisitely indicate how investigations of rare families provide novel insights with profound implications for understanding gene-gene interactions and elucidating the sequential roles of respective gene products in specific metabolic pathways.

When family members are assessed, we also observed that 85.7% of *CETP* mutation carriers assessed in this study have HDLc≥90^th^ percentile, compared to 66.3% of individuals with *LIPG* mutations and only 50.0% with *GALNT2* mutations. Conversely, only one individual with *CETP* mutations had HDLc between 80–89^th^ percentiles, compared to 10.8% of individuals with *LIPG* mutations and 40.0% with *GALNT2* mutations. These observations may imply differential effects on function for mutations in each of these genes. Computational assessment predicts damaging effects of all of these DNA changes on function but clearly functional studies are warranted to clarify this. Alternatively, these findings are also compatible with the concept that defects in cholesterol ester unloading from HDL may confer larger effects on HDLc elevation.

A total of 6 of 10 mutations identified in *LIPG*, *CETP*, and *GALNT2* were novel. These mutations are likely to reduce protein activity and lead to elevated HDLc in humans based on the following. First, novel mutations largely occurred in highly conserved amino acid residues and/or were predicted to cause premature protein truncation or damage protein function. Second, they were detected in 8 of 171 probands with HDLc≥90^th^ percentile (4.7%) but none of 136 probands with HDLc≤10^th^ percentile (e.g., p = 9.9*10^−3^ by CAST; Table 2). Third, they segregated with elevated HDLc in family members of probands identified with mutations. These observations indicate that the majority of novel mutations described here are likely deleterious to protein function, although further validation awaits *in vitro* functional analysis.

Where data are available, the lipid phenotypes of individuals with mutations in this study bear a striking similarity to drug effects observed from emerging clinical trials. For example, individuals with heterozygous *CETP* mutations and expected 50% activity in this study had 62.3% increased HDLc and 26.4% reduced LDLc compared to first-degree relative controls (Table 4). These studies would predict that further inhibition should enhance these effects. Indeed, patients treated with anacetrapib, a CETP inhibitor that reduces CETP activity by ∼90% [44], had 138.1% increased HDLc and 39.8% reduced LDLc when compared to individuals treated with placebo [9], or roughly 1.5–2.5 times the effects observed with heterozygous, loss-of-function *CETP* mutations. Our results therefore suggest that detailed analysis of patients with rare, loss-of-function mutations may be a useful predictor of expected lipid changes with drugs conferring significant loss of function in this gene.

The following limitations of our study should be considered. First, the relatively small size of our initial proband cohort with a focus exclusively on Dutch-Caucasian individuals indicates that mutation frequencies and effects on HDLc elevation should be investigated in independent groups, and ideally in very large populations of Dutch or similar ancestries. This is particularly necessary to establish population-based mutation frequencies for *CETP* and *GALNT2* mutations, as our cohort is underpowered to detect statistically significant enrichment of these rare events with elevated HDLc in unrelated probands. Second, our analyses do not correct for the effects of multiple testing. For example, if Bonferroni correction for the multiple comparisons of HDLc elevation in mutation carriers vs. first-degree relative controls across all genes is considered, a significant p value threshold should be 0.05/3 = 0.017, thereby making our observation of *CETP* mutation segregation with elevated HDLc borderline significant. However, while the Bonferroni correction is a robust but conservative method that can underestimate statistical significance, these observations further emphasize the need for additional assessment of these and other mutations in independent cohorts and families. Third, our sequence analysis is limited to the *LIPG*, *CETP*, and *GALNT2* coding regions and adjacent sequence. Notably, a recent study identified rare gain-of-function promoter mutations in *LIPG* in individuals with low HDLc [22]. It is therefore of interest to determine whether rare gain-of-function mutations in the gene promoters may also underlie low HDLc in this cohort. Finally, while our human genetics-based approach successfully identifies exceedingly rare individuals with *LIPG* mutations plus mutations in *ABCA1*, *LCAT*, and *LPL*, in addition to *LIPG* plus *GALNT2* mutations, these observations should be assessed in additional individuals, which may now be more readily enabled by the advent of next-generation sequencing technologies [45]. For example, exon capture technologies combined with next-generation sequencing can be used to simultaneously assess multiple genes that underlie HDLc levels in numerous probands [46], thereby increasing the probability of identifying individuals with more than one mutation. Alternatively, in probands with *LIPG* mutations and low HDLc, exome or genome sequencing may identify new mutations in addition to those already found in *ABCA1*, *LCAT*, and *LPL*.

In conclusion, we show that *LIPG*, *CETP*, and *GALNT2* mutations are preferentially found in individuals with HDLc≥90^th^ percentile and segregate with elevated HDLc in families. We also show that some *LIPG* mutations do not overcome the deleterious effects of *ABCA1*, *LCAT*, and *LPL* mutations. Moreover, we indicate that *GALNT2* mutations do not further elevate HDLc in individuals with *LIPG* mutations, indicating that mutations in these genes may be redundant to HDLc elevation. These studies provide a foundation by which to assess the effects of *LIPG*, *CETP*, and *GALNT2* mutations on CAD risk and the potential of these genes to serve as effective targets for drug development. Finally, we show that additional causes of elevated HDLc in this cohort are yet to be discovered.

## Supporting Information

Figure S1 **Segregation of** ***LIPG*** **,** ***CETP*** **, and** ***GALNT2*** **mutations with elevated HDLc in pedigrees.** For each individual, the individual ID, Age (in years), total cholesterol (mmol/L), triglycerides (mmol/L), HDLc (in mmol/L) plus [HDLc percentile], BMI, and genotype(s) for listed mutations are shown. Squares, Males; Circles, Females; Arrow, proband. Filled shape, HDLc≥90^th^ percentile; half-filled, HDLc between 80–89^th^ percentiles; empty shape, HDLc<80^th^ percentile. Three probands for which no pedigree data are available are also shown.
(TIF)

Figure S2 **Segregation of** ***LIPG*** **, mutations in pedigrees with** ***ABCA1*** **,** ***LCAT*** **, and** ***LPL*** **mutations.** Data are presented as described in Figure S1, except filled shape, HDLc≤10^th^ percentile; half-filled, HDLc between 10–20^th^ percentiles; empty shape, HDLc>20^th^ percentile.
(TIF)

Table S1 **Additional** ***GALNT2*** **SNPs found in 55 probands with HDLc≥90^th^ vs. 55 probands with HDLc<10^th^ percentiles.** P values are calculated by Fisher's exact test.
(DOC)

Table S2 **Additional** ***LIPG*** **and** ***CETP*** **SNPs found in 55 probands with HDLc≥90^th^ vs. 55 probands with HDLc≤10^th^ percentiles.** P values are calculated by Fisher's exact test.
(DOC)

Table S3 **Phenotypes of individuals with** ***LIPG+ABCA1*** **mutations in families.**
(DOC)

Table S4 **Phenotypes of individuals with** ***LIPG+LCAT*** **mutations in families.**
(DOC)

Table S5 **Phenotypes of individuals with** ***LIPG+LPL*** **mutations in families.**
(DOC)

Table S6 **Association of** ***LIPG+GALNT2*** **mutations with HDLc in families.**
(DOC)

Table S7 **PCR primer sequences.**
(DOC)
