# Supplementary figures and images for: Segregation of LIPG, CETP, and GALNT2 Mutations in Caucasian Families with Extremely High HDL Cholesterol
Source: PLoS One. 2012 Aug 27;7(8):e37437. doi: 10.1371/journal.pone.0037437 (PMC3428317; doi:10.1371/journal.pone.0037437)

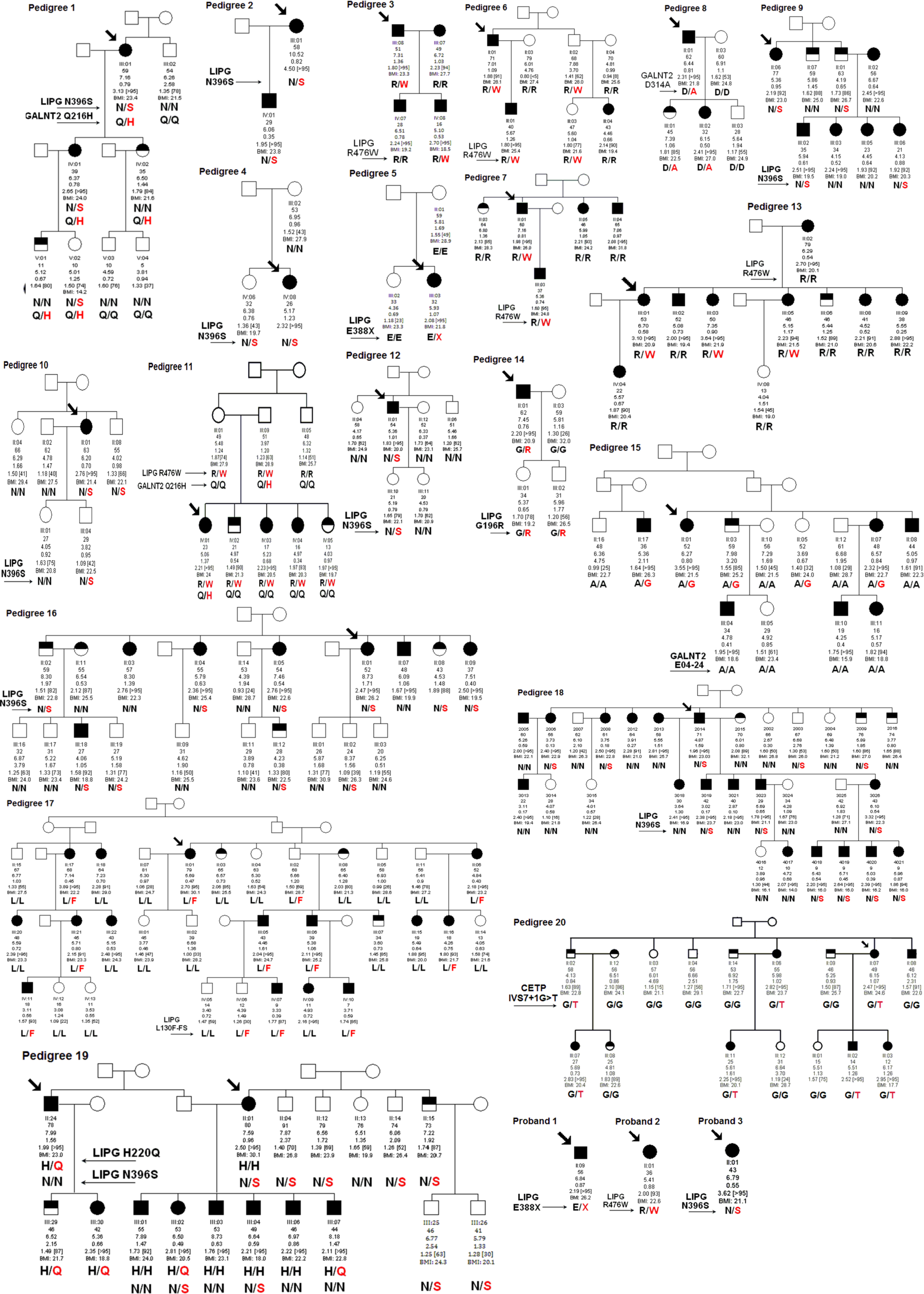

Supplement: Figure S1 — Segregation of LIPG , CETP , and GALNT2 mutations with elevated HDLc in pedigrees. For each individual, the individual ID, Age (in years), total cholesterol (mmol/L), triglycerides (mmol/L), HDLc (in mmol/L) plus [HDLc percentile], BMI, and genotype(s) for listed mutations are shown. Squares, Males; Circles, Females; Arrow, proband. Filled shape, HDLc≥90th percentile; half-filled, HDLc between 80–89th percentiles; empty shape, HDLc<80th percentile. Three probands for which no pedigree data are available are also shown. (TIF) [file pone.0037437.s001.tif]

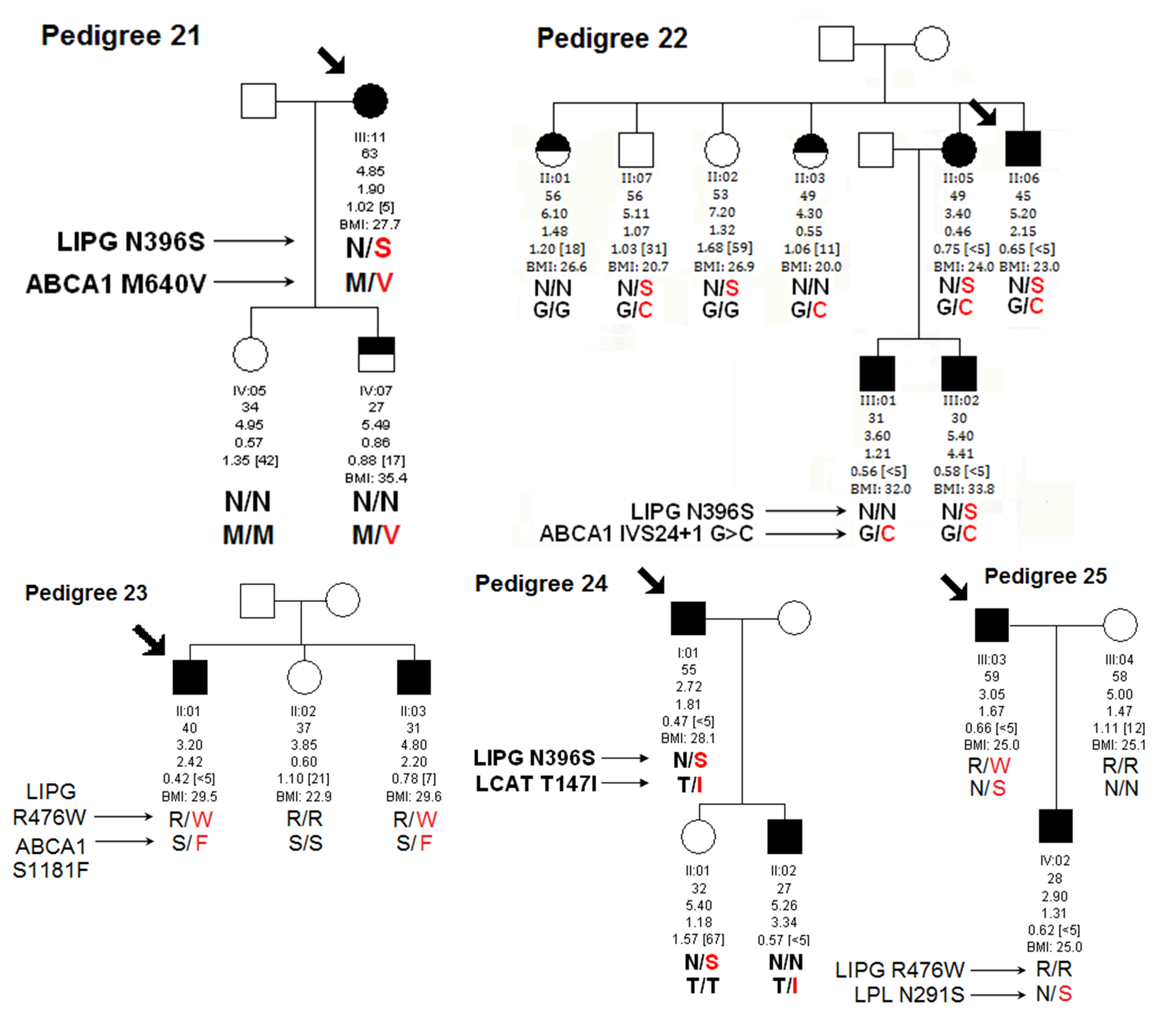

Supplement: Figure S2 — Segregation of LIPG , mutations in pedigrees with ABCA1 , LCAT , and LPL mutations. Data are presented as described in Figure S1, except filled shape, HDLc≤10th percentile; half-filled, HDLc between 10–20th percentiles; empty shape, HDLc>20th percentile. (TIF) [file pone.0037437.s002.tif]
