# Supplementary material for: Segregation of LIPG, CETP, and GALNT2 Mutations in Caucasian Families with Extremely High HDL Cholesterol
Source: PLoS One. 2012 Aug 27;7(8):e37437. doi: 10.1371/journal.pone.0037437 (PMC3428317; doi:10.1371/journal.pone.0037437)
Supplement: Table S1 — Additional GALNT2 SNPs found in 55 probands with HDLc≥90th vs. 55 probands with HDLc<10th percentiles. P values are calculated by Fisher's exact test. (DOC) [file pone.0037437.s003.doc]

Table S1. Additional *GALNT2* SNPs found in 55 probands with HDLc ≥90^th^ vs. 55 probands with HDLc <10^th^ percentiles. P values are calculated by Fisher’s exact test.

| GALNT2 SNPs | Chr1 Position (hg18) | Class | Effect | Major Allele | Minor Allele | MAF (HDLc ≥90th %ile) | MAF (HDLc <10th %ile) | p value |
| --- | --- | --- | --- | --- | --- | --- | --- | --- |
| rs678050 | 228,380,534 | Intronic | None (Spliceview) | T | C | 0.10 | 0.14 | 0.532 |
| Novel SNP | 228,438,467 | Synonymous | Leu153Leu | C | T | 0.00 | 0.01 | 1.000 |
| rs3811488 | 228,438,506 | Intronic | None (Spliceview) | C | T | 0.38 | 0.44 | 0.493 |
| rs3811487 | 228,438,648 | Intronic | None (Spliceview) | T | C | 0.04 | 0.07 | 0.374 |
| rs3811486 | 228,438,684 | Intronic | None (Spliceview) | T | C | 0.06 | 0.05 | 1.000 |
| rs34897003 | 228,438,740 | Synonymous | Phe164Phe | G | A | 0.01 | 0.02 | 1.000 |
| rs1923950 | 228,451,593 | Synonymous | Thr286Thr | G | A | 0.07 | 0.09 | 0.807 |
| rs2273965 | 228,452,797 | Intronic | None (Spliceview) | C | T | 0.05 | 0.05 | 1.000 |
| Novel SNP | 228,452,803 | Intronic | None (Spliceview) | C | T | 0.01 | 0.00 | 1.000 |
| rs2273966 | 228,452,964 | Intronic | None (Spliceview) | T | A | 0.28 | 0.32 | 0.659 |
| rs3811485 | 228,465,068 | Intronic | None (Spliceview) | C | T | 0.06 | 0.05 | 1.000 |
| rs12091838 | 228,476,785 | Intronic | None (Spliceview) | C | T | 0.12 | 0.16 | 0.439 |
| rs2273970 | 228,481,771 | Nonsynonymous | Val554Met (Polyphen: Benign) | C | T | 0.04 | 0.04 | 1.000 |
| rs2273968 | 228,481,867 | 3' UTR | Unknown | A | G | 0.13 | 0.06 | 0.167 |
| rs2273967 | 228,481,916 | 3' UTR | Unknown | G | A | 0.27 | 0.24 | 0.643 |
