# Supplementary material for: Segregation of LIPG, CETP, and GALNT2 Mutations in Caucasian Families with Extremely High HDL Cholesterol
Source: PLoS One. 2012 Aug 27;7(8):e37437. doi: 10.1371/journal.pone.0037437 (PMC3428317; doi:10.1371/journal.pone.0037437)
Supplement: Table S2 — Additional LIPG and CETP SNPs found in 55 probands with HDLc≥90th vs. 55 probands with HDLc≤10th percentiles. P values are calculated by Fisher's exact test. (DOC) [file pone.0037437.s004.doc]

Table S2. Additional *LIPG* and *CETP* SNPs found in 55 probands with HDLc ≥90^th^ vs. 55 probands with HDLc ≤10^th^ percentiles. P values are calculated by Fisher’s exact test.

| LIPG SNPs | Chr18 Position (hg18) | Class | Effect | Major Allele | Minor Allele | MAF (HDLc ≥90th %ile) | MAF (HDLc <10th %ile) | p value |
| --- | --- | --- | --- | --- | --- | --- | --- | --- |
| rs874566 | 45,342,739 | Synonymous | Ser21Ser | C | T | 0.00 | 0.01 | 1.000 |
| rs2000813 | 45,347,862 | Nonsynonymous | Thr111Ile (Polyphen: Benign) | C | T | 0.33 | 0.24 | 0.177 |
|  |  |  |  |  |  |  |  |  |
| CETP SNPs | Chr16 Position (hg18) | Class | Effect | Major Allele | Minor Allele | MAF (HDLc ≥90th %ile) | MAF (HDLc <10th %ile) | p value |
| rs5883 | 55,564,854 | Synonymous | Phe287Phe | C | T | 0.08 | 0.03 | 0.135 |
| rs5882 | 55,573,593 | Nonsynonymous | Ile422Val (Polyphen: Benign) | A | G | 0.40 | 0.29 | 0.119 |
