# Supplementary material for: Segregation of LIPG, CETP, and GALNT2 Mutations in Caucasian Families with Extremely High HDL Cholesterol
Source: PLoS One. 2012 Aug 27;7(8):e37437. doi: 10.1371/journal.pone.0037437 (PMC3428317; doi:10.1371/journal.pone.0037437)
Supplement: Table S3 — Phenotypes of individuals with LIPG+ABCA1 mutations in families. (DOC) [file pone.0037437.s005.doc]

| Table S3. Phenotypes of individuals with *LIPG + ABCA1* mutations in families. | | | | | |
| --- | --- | --- | --- | --- | --- |
| Measure | Mutation carriers | | | |  |
|  | LIPG + ABCA1 | LIPG | p value | ABCA1 | p value |
| Total assessed | 7 | 83 |  | 93 |  |
| Age (y) ^a^ | 44.9 (12.3) | 43.9 (20.7) | 0.903 | 49.2 (14.3) | 0.439 |
| Male individuals ^b^ | 5 (71.4%) | 46 (55.4%) | 0.417 | 41 (44.1%) | 0.165 |
| Total cholesterol (mmol/L) ^a^ | 4.57 (0.89) | 5.83 (1.41) | 0.023 | 4.55 (1.27) | 0.971 |
| Triglycerides (mmol/L) ^a^ | 2.09 (1.24) | 1.02 (0.64) | 2.06*10^-4^ | 1.86 (1.63) | 0.716 |
| HDLc (mmol/L) ^a^ | 0.75 (0.22) | 2.04 (0.64) | 9.77*10^-7^ | 0.70 (0.32) | 0.681 |
| LDLc (mmol/L) ^a^ | 2.86 (0.67) | 3.32 (1.21) | 0.325 | 2.99 (1.18) | 0.761 |
| BMI (kg/m2) ^a^ | 26.9 (4.6) | 22.7 (3.2) | 2.19*10^-3^ | 27.1 (6.1) | 0.943 |
| ^a^, Average (SD) shown; ^b^, N (%) shown | | | | | |
