# Supplementary material for: Segregation of LIPG, CETP, and GALNT2 Mutations in Caucasian Families with Extremely High HDL Cholesterol
Source: PLoS One. 2012 Aug 27;7(8):e37437. doi: 10.1371/journal.pone.0037437 (PMC3428317; doi:10.1371/journal.pone.0037437)
Supplement: Table S4 — Phenotypes of individuals with LIPG+LCAT mutations in families. (DOC) [file pone.0037437.s006.doc]

| Table S4. Phenotypes of individuals with *LIPG + LCAT* mutations in families. | | | |
| --- | --- | --- | --- |
| Measure | Mutation carriers | | |
|  | LIPG + LCAT | LIPG | LCAT |
| Total assessed | 1 | 83 | 66 |
| Age (y) ^a^ | 55 | 43.9 (20.7) | 44.8 (14.1) |
| Male individuals ^b^ | 1 | 46 (55.4%) | 44 (66.7%) |
| Total cholesterol (mmol/L) ^a^ | 2.72 | 5.83 (1.41) | 4.61 (1.05) |
| Triglycerides (mmol/L) ^a^ | 1.81 | 1.02 (0.64) | 1.51 (0.68) |
| HDLc (mmol/L) ^a^ | 0.47 | 2.04 (0.64) | 0.72 (0.23) |
| LDLc (mmol/L) ^a^ | 1.42 | 3.32 (1.21) | 3.19 (0.96) |
| BMI (kg/m2) ^a^ | 28.1 | 22.7 (3.2) | 25.5 (4.4) |
| ^a^, Average (SD) shown; ^b^, N (%) shown | | | |
