# Supplementary material for: Segregation of LIPG, CETP, and GALNT2 Mutations in Caucasian Families with Extremely High HDL Cholesterol
Source: PLoS One. 2012 Aug 27;7(8):e37437. doi: 10.1371/journal.pone.0037437 (PMC3428317; doi:10.1371/journal.pone.0037437)
Supplement: Table S5 — Phenotypes of individuals with LIPG+LPL mutations in families. (DOC) [file pone.0037437.s007.doc]

| Table S5. Phenotypes of individuals with *LIPG + LPL* mutations in families. | | | |
| --- | --- | --- | --- |
| Measure | Mutation carriers | | |
|  | LIPG + LPL | LIPG | LPL |
| Total assessed | 1 | 83 | 13 |
| Age (y) ^a^ | 59 | 43.9 (20.7) | 42.9 (12.9) |
| Male individuals ^b^ | 1 | 46 (55.4%) | 10 (78.6%) |
| Total cholesterol (mmol/L) ^a^ | 3.05 | 5.83 (1.41) | 4.00 (1.22) |
| Triglycerides (mmol/L) ^a^ | 1.67 | 1.02 (0.64) | 1.54 (0.89) |
| HDLc (mmol/L) ^a^ | 0.66 | 2.04 (0.64) | 0.67 (0.25) |
| LDLc (mmol/L) ^a^ | 1.62 | 3.32 (1.21) | 2.62 (1.23) |
| BMI (kg/m2) ^a^ | 25 | 22.7 (3.2) | 28.9 (5.7) |
| ^a^, Average (SD) shown; ^b^, N (%) shown | | | |
