# Supplementary material for: Segregation of LIPG, CETP, and GALNT2 Mutations in Caucasian Families with Extremely High HDL Cholesterol
Source: PLoS One. 2012 Aug 27;7(8):e37437. doi: 10.1371/journal.pone.0037437 (PMC3428317; doi:10.1371/journal.pone.0037437)
Supplement: Table S6 — Association of LIPG+GALNT2 mutations with HDLc in families. (DOC) [file pone.0037437.s008.doc]

| Table S6. Association of *LIPG + GALNT2* mutations with HDLc in families. | | | | | | | |
| --- | --- | --- | --- | --- | --- | --- | --- |
| Measure | Mutation carriers | | | | | | |
|  | LIPG + GALNT2 | 1st-degree relative controls | p value | LIPG | p value | GALNT2 | p value |
| Total assessed | 5 | 11 |  | 83 |  | 10 |  |
| Age (y) ^a^ | 36.4 (20.0) | 31.5 (18.3) | 0.639 | 43.9 (20.7) | 0.433 | 43.2 (15.1) | 0.473 |
| Male individuals ^b^ | 1 (20.0%) | 8 (72.7%) | 0.053 | 46 (55.4%) | 0.126 | 4 (40.0%) | 0.475 |
| Total cholesterol (mmol/L) ^a^ | 5.51 (1.25) | 5.25 (0.95) | 0.646 | 5.83 (1.41) | 0.631 | 6.15 (1.20) | 0.360 |
| Triglycerides (mmol/L) ^a^ | 1.08 (0.27) | 1.38 (1.30) | 0.615 | 1.02 (0.64) | 0.854 | 1.21 (0.84) | 0.742 |
| HDLc (mmol/L) ^a^ | 2.14 (0.79) | 1.52 (0.29) | 0.032 | 2.04 (0.64) | 0.726 | 2.04 (0.64) | 0.791 |
| LDLc (mmol/L) ^a^ | 2.87 (0.67) | 3.09 (0.75) | 0.583 | 3.32 (1.21) | 0.420 | 3.55 (1.02) | 0.208 |
| BMI (kg/m2) ^a^ | 22.9 (5.3) | 21.4 (3.1) | 0.521 | 22.7 (3.2) | 0.922 | 23.6 (2.1) | 0.720 |
| ^a^, Average (SD) shown; ^b^, N (%) shown | | | | | | | |
