# Supplementary material for: Segregation of LIPG, CETP, and GALNT2 Mutations in Caucasian Families with Extremely High HDL Cholesterol
Source: PLoS One. 2012 Aug 27;7(8):e37437. doi: 10.1371/journal.pone.0037437 (PMC3428317; doi:10.1371/journal.pone.0037437)
Supplement: Table S7 — PCR primer sequences. (DOC) [file pone.0037437.s009.doc]

| Table S7. PCR primer sequences. | | | |
| --- | --- | --- | --- |
| Gene | Exon | Forward sequence | Reverse sequence |
| ABCA1 | 1 | CGTGCTTTCTGCTGAGTGAC | GATCAAAGTCCCCGAAACC |
|  | 2 | GGCTGGATTAGCAGTCCTCA | ATCCCCAACTCAAAACCACA |
|  | 3 | GGATTTCCCAGATCCCAGTG | AAGTCCAATTTAGCCCACGTT |
|  | 4 | GACAGACTTGGCATGAAGCA | CCAGCCATTCAAAATTCTCC |
|  | 5 | GCACTTGGCAGTCACTTCTG | GGTGCAGGTCAATTTCCAAT |
|  | 6 | CGTTTCTCCACTGTCCCATT | CCCCTTCACCACCATTACAA |
|  | 7 | ACTTCAAGGACCCAGCTTCC | TGTCCAAGGAAAAGCCTCAC |
|  | 8 | TCGGTTTCTTGTTTGTTAAACTCA | AGGACCTCTTGCCAGACTCA |
|  | 9 | TCCCAAGGCTTTGAGATGAC | AGGAGATGACACAGGCCAAG |
|  | 10 | GGCTCCAAAGCCCTTGTAA | CGCACACCTCTGAAGCTACC |
|  | 11 | GCTGCTGTGATGGGGTATCT | ACCTCACTCACACCTGGGAA |
|  | 12 | TTTGTAAATTTTGTAGTGCTCCTCA | GCCTCCTGCCTGAACCTTAT |
|  | 13 | TAGTCAGCCCTTGCCTCCTA | CAAAATCATGACACCAAGTTGAG |
|  | 14 | AAAGGGGCTTGGTAAGGGTA | CATGCACATGCACACACATA |
|  | 15 | GATGTGGTGCTCCCTCTAGC | CCTTAGCCCGTGTTGAGCTA |
|  | 16 | CAAGTGAGTGCTTGGGATTG | TGCTTTTATTCAGGGACTCCA |
|  | 17 | GCAAATTCAAATTTCTCCAGG | CCCATGCACTGCAGAGATTC |
|  | 18 | TCAAGGAGGAAATGGACCTG | AAGGCAGGAGACATCGCTT |
|  | 19 | CTGAAAGTTCAAGCGCAGTG | GGGATCAGCATGGTTTCCTA |
|  | 20 | TGCAGACTGAATGGAGCATC | GCTTAAGTCCCACTCCTCCC |
|  | 21 | GCCAGGGGACACTGTATTCT | ATTTTCCTCCGCATGTGTGT |
|  | 22 | AGGTCCTCTGCCTTCACTCA | TCACAGAAGCCTAGCCATGA |
|  | 23 | CCAGTGCTTACCCCTGCTAA | AACAGAGCAGGGAGATGGTG |
|  | 24 | CACACAACAGAGCTTCTTGGA | TCTGCACCTCTCCTCCTCTG |
|  | 25 | ACCTGGAACAGGTGTGGTGT | ACTGGGGCCAACATTAATCA |
|  | 26 | GGGCTAACATGCCACTCAGTA | CTTCCCCATCTGCAACAAAC |
|  | 27 | GTTTGTTGCAGATGGGGAAG | GCTAAAGGCCATCCAAAGAA |
|  | 28 | CACCAGAAGAAGGAGCATGG | TCAAGTGCATCTGGGCATAA |
|  | 29 | CTGGACTCGTAGGGATTTGC | TCTGAAGTCCATTCCCTTGG |
|  | 30 | GCCTGTCACAGAGAAATGCTT | CAATGTGGCATGCAGTTGAT |
|  | 31 | TTACGGAATGATCCTGTGCTC | GAAGCTACCAGCCCATCCT |
|  | 32 | AGTCAGGTTTCCGGTCACAC | CATTTCCCCCACTGTTTCAG |
|  | 33 | CCGTTCCTTATATCCTCAGGTG | CCAAGGCTTTCTTCAATCCA |
|  | 34 | CCTTGTACACACTCGCACTGA | GATCCGTTTAACCTGCCAAC |
|  | 35 | TGTTGTCCACAGGTTCCAGA | ATGCCCCTGCCAACTTTAC |
|  | 36 | TGAGGTTTATGGGCATGGTT | CTCTGCAGCTGTTCCCCTAC |
|  | 37 | ATGTTTTTCCTTGGCTGTGC | TATCAATCCATGGCCCTGAC |
|  | 38 | ATCTGCCCTTTCTTGTCTGA | AGAGTCCCTGCCCTCCTTCT |
|  | 39 | AGGGAGCTGCACAGTGGATA | AAGGCAGTCAGCAGTGTCAA |
|  | 40 | TCACTCCCATATTTCAGAACTTGA | GGGGAACATCCTGTGCTTAG |
|  | 41 | TGTTTATTGGAAGATCGGTGAA | CCATTGGTGAGTGTTTCCCT |
|  | 42 | CGTTAGAGACTGAATCTTTGTCCTG | AGTCAGCAAACTGCTGGGTT |
|  | 43 | AGTCCTGCCTTCCACAGTTG | ATTGCTCCATCCTGGCATAA |
|  | 44 | GGTAGTTACGTGTTAGGGGCA | TCATGGATGATTTTATGTGCTTC |
|  | 45 | CAGGAACATTAGGCCAGATTG | GCGTGTGGAAAAGCCATAAG |
|  | 46 | CATGTATGTGTAGGACAGCATGA | GCCAATCATACAACAGCCCT |
|  | 47 | CTGTTTCAAAGATGCTTCTGC | TGATCGCATATTCTACTTGGAAA |
|  | 48 | CAGCATCATCCCTATATCCACA | CCCTTTATTTTAGAGGCACCA |
|  | 49 | GGGTTCCCAGGGTTCAGTAT | GATCAGGAATTCAAGCACCAA |
|  | 50 | CTTGACCTAATTTCAACATCTGG | TGGGTTCCATAATAGAGTTTCACA |
|  |  |  |  |
| APOA1 | 1 | AAGTTCCACATTGCCAGGAC | ACAGAGCGGGAGAAGACCTC |
|  | 2 | CTCTGTGCCCTTCTCCTCAC | GTGAGAAACCTGCTGCCTCT |
|  | 3 | TCAGATCTCAGCCCACAGC | CCAGTCTGGCTTCAACATCA |
|  | 4 | CTGGAAATGCTAGGCCACTG | CAGCTCGTCGCTGTAGGG |
|  | 4 | AAGAGAAGCTGAGCCCACTG | AAGCTGCTTCCCACTTTGG |
|  |  |  |  |
| CETP | 1 | AGGGGAGACAAGTAGAAGTTGG | GCACTCCTCTCTCTGGGCTA |
|  | 2 | CCAACCCCTCAGCTTTAAGA | AGGCTCTGGTTCCAAATCAA |
|  | 3-5 | CCTCGCCTAGACAAAATTGG | CCCCACTGAAGGTATTGCTG |
|  | 6-7 | TCGTGTGTGTGACAGGTGTG | ACAGTCCCAAGGCCACATAG |
|  | 8 | GAGGGTTGGGTAGCTGTGTG | ACTCTTGGCTGCCATAAGGTT |
|  | 9 | GTTTCCCCATCTGCACTCTG | CACCACCAAGTTTCCGAGTT |
|  | 10 | GGTCCAGTCCTTGAAACTGC | TGCCATTGGTGGTGTTATTG |
|  | 11 | CAGCTTTGTCCTTCCCATCT | GGTGGTGAGAAGGATCTGGA |
|  | 12 | CCCGTGTCATACTTGCCTCT | CACATTTCCCCACCAACAC |
|  | 13 | AGGGCCTGAGCTATGAGACA | TCTGGTTGCCTGATTTCCTT |
|  | 13 | CTCAAGAGAGTGCCCCAAAG | GACCACCTGGTCCACATTCT |
|  | 14 | TGAGGATGAATGCTTGTCCA | GCCAGGACCGCTAAGTATCA |
|  | 15 | CAGGCAAACTCTGCTCTTCC | GAGGGAGCCAAGCTGGTAG |
|  | 16 | CTTGGCTCCCTCCTGGTG | GACGGCACTGGTGAGACAAT |
|  |  |  |  |
| GALNT2 | 2 | CTCCTGCTCATCAGTGCATC | CTCCAAGAGCCATGCTCAGT |
|  | 3 | TGCAGTCTCTGTGATGAGCA | TCACAGGTGAAAGCGACGTA |
|  | 4 | CCATCCCAGTTGGTCAGTCT | GACAGCCCTGGTCTGACTGT |
|  | 5 | TGTAGCTCCTCCAACCAAGG | CCTCATTAGCACCCCCACTA |
|  | 6 | CGGCCCTTAGATGATTGAGA | GGCTCTGGTAGAGGGAGACA |
|  | 7 | CAGAAAGCAGGCTGCAGAG | TCCCTTCTGTCCCCTCTACC |
|  | 8 | CCCTGTGCCTGCCTAATACT | CACACTACGGCGTGAACACT |
|  | 9 | CTGTGAGGAATGGGGGTGT | GAGCTGAGACGAGGGGAAAC |
|  | 10 | AAGGGTGCTGGCAATCTAAC | GGATCGATTAAGCCCAGTGA |
|  | 11 | TACCTGCTTGGTGTGTCTGC | AAGGTCAGGTCCTGCTTTCA |
|  | 12 | CAGGTGCAAATGGAGTGATG | GGCACCTGGGTAGCTGAAT |
|  | 13 | CAACCCTGTTCTCCTCAGCA | TTTCCCCTCTGCTGCTTCTA |
|  | 14 | CATGAGGCCACTGAGCAAAG | GGCAGCTGGGACTTCTAACA |
|  | 15 | GATGCCCCTTCTTTCCTTTC | TACACGCAGAGATGGATGCT |
|  | 16 | TCCTGAATTCACACGAATCTG | TGAAAGCTGGAAGGAAGAGG |
|  |  |  |  |
| LCAT | 1 | GCAATCTCTGGCCACAACC | TTATGTCGGGGCTTATGCAG |
|  | 2 | GACGGGGGTAAGGGTCAC | GCCACTCAGCAGCCAGTAG |
|  | 3 | TTGGCTACTGGCTGCTGAGT | GCCATTGTTGACCAGGTTCT |
|  | 4 | TGTGGAGTACCTGGACAGCA | AAGACAGGCTTCCCATAGGC |
|  | 5 | GGTGAGTGTCTCTGCGGATG | GGCCACTGTGAGCAGGAG |
|  | 6 | GTCTAGATTGGGCAGGGACA | CACCGTGTCATCACCATCC |
|  | 6 | GCCCCGCACCTACATCTAC | GAGCCTGTGGCTGGTGAG |
|  |  |  |  |
| LIPG | 1 | AGATCTCGTTCTGGGGCAAG | GGAGGGTTTGAAGAATCAGC |
|  | 2 | TCAGACCCCAGGTCTCTCAC | AATACAAGCAGCCCACAAGG |
|  | 3 | GCAACAGAGTGAGACCCTGA | CATGGCAACAGATGCATGAC |
|  | 4 | TGTAGCATCACCAGCAAAGC | ATGCTTTCCAGGTTCCTTCC |
|  | 5 | TCATCTTCATTCTGCACACTCAG | TGCTAAAGGCTCTGGCTGAT |
|  | 6 | CTGGGCAAGAGTGAGACCTT | CTGCTCTGCAGCATGGTATT |
|  | 7 | CCAAAAGAACACCAGCCCTA | AGCTAGACCACCTGGCTCCT |
|  | 8 | GTGGGGAAAGCACTCACACT | ATGCTGGAGGGAAGGATTG |
|  | 9 | GGGGAATTCTGAAGGCTTTT | CGGCCCAACTTCTTAAAGG |
|  | 10 | GTCAAAAGAGCACACCCTGA | CCCAGCTGCTTACACACAAA |
|  |  |  |  |
| LPL | 2 | CTCATATCCAATTTTTCCTT | CTCTTCCCCAAAGAGCCTCC |
|  | 5 | TGTTCCTGCTTTTTTCCCTT | TAATTCGCTTCTAAATAATA |
|  | 6 | GCCGAGATACAATCTTGGTG | GCATGATGAAATAGGACTCC |
|  | 9 | TATTCACATCCATTTTCTTC | GTCAGCTTTAGCCCAGAATG |
